# How social and political participation in later life divergently impact on perceived health of older adults: a case study in Central Italy

**DOI:** 10.3389/fpubh.2026.1901328

**Published:** 2026-08-24

**Authors:** Mario Cantore, Sara Santini, Marco Socci

**Affiliations:** Center for Socio-Economic Research on Aging, IRCCS INRCA-National Institute of Health and Science on Aging, Ancona, Italy

**Keywords:** civic participation, health, intergenerational dialogue, older adults, political participation, social participation, wellbeing

## Abstract

In an ever-evolving aging society, civic participation, including social and political engagement, can have positive effects on older adults’ health and wellbeing, according to the Acitve Aging framework. This study aimed at understanding the experience of civic participation of a group of Italian older adults, the perceived health outcomes and how social and political participation divergently impact health and wellbeing in later life. In 2025, 12 older adults living in Central Italy and aged 70 and over were asked to answer a semi-structured interview. The contents were transcribed verbatim and thematically analyzed. Civic participation makes interviewees feel mentally and physically active and useful in the community. Most of them have been socially and/or politically engaged since they were younger. For someone retirement disclosed new opportunities for civic involvement. While voluntary participation promoted respondents’ physical mobility, agency, and self-efficacy, political participation reveals a marked ambivalence: serving as a cognitive stimulus on one hand, but provoking emotional fatigue, alienation, and frustration on the other. Intergenerational learning and dialogue for strengthening civic participation in the communities is a common thought. The findindgs highlight the importance of avoiding a one-size-fits-all approach for promoting civic participation among older adults in Europe by taking into consideration the possible emotional distress that can come from political engagement. Frustration and stress coming from the disillusionment in policy and society can be overcome by involving older adults in co-participatory intergenerational stakeholders’ consultation tools, with the aim of informing evidence-based Active Aging public policies and health promotion strategies.

## 1 Introduction

Participation in the community through civic and political activities is named as civic participation or civic engagement in the literature. Civic participation is commonly defined as “the active involvement of citizens in community life, with the aim of improving the conditions of others or contributing to shaping the community’s future” (1) and it encompasses both social and political participation (2). Recently, Serrat (3) defined civic engagement as a “a multidimensional concept that encompasses a diverse range of activities through which older adults contribute to their families, communities and society at large” e.g. informal help within and outside the family, associational membership, formal volunteering, institutionalised and non- institutionalised political activities.

In this paper, we refer to civic engagement and civic participation as synonimous, focusing only on social and political participation as defined below, omitting the provision of supporting behaviors to family members and/or friends, e.g., informal caregiving.

Social participation is considered one of the most important determinants of health in later life and it consists on interpersonal interactions outside home environment (i.e., in the commmunity and in the society) and the satisfaction with these interactions (4). Depending on the type of resources shared, it can take three forms: collective, where most time is shared (e.g., travel groups); productive, where the interaction results in services that benefit the community (e.g., volunteering); and political, where decisions are made and funds are allocated for the benefit of a group (5). In a nutshell, social participation can be defined as the result of community-based activities, interpersonal interactions, resource sharing, active participation and individual satisfaction (6).

Political participation includes voting, supporting a political campaign, donating money to a candidate or a cause, contacting politicians, but also any non-electoral modes of participation, e.g., engaging in political organisations and social movements, expressions of dissent, protesting (7, 2).

According to Huntington and Nelson (8), political participation is understood as an activity undertaken by private citizens to influence government decision-making either directly, by shaping the formulation or implementation of public policies, or indirectly, by affecting the selection of individuals responsible for policy-making (9).

Furthemore, contemporary authors distinguish political participation into institutionalised and non-institutionalised organisations, intending the former as regular established political bodies and the second one as not structured movements promoting petitions and boycotting activities (10).

In the following paragraphs the main theoretical perspectives on social and political participation in later life are provided as well as the impact of such an engagement on older adults’ health and wellbeing.

### 1.1 Social and political participation in later life

Understanding civic participation in later life requires engagement with a broad set of sociological and life-course theories that explain how individuals adapt to aging and how structural factors shape their engagement within the society.

Early theoretical perspectives emphasized normative patterns of aging, with two foundational but contrasting approaches: Activity Theory (AT) and Disengagement Theory (DT). AT (11, 12) posits that wellbeing in older age is strongly linked to maintaining social roles, activities, and interactions, suggesting that participation—whether civic, community, or political—supports identity continuity and psychological health. In contrast, DT (13) proposes that aging involves a gradual, reciprocal withdrawal between individuals and society, in which reduced participation is viewed as a natural adjustment that benefits both the individual and the social system. Subsequent critiques have questioned its universality and applicability across different cultural and socioeconomic contexts (14), yet its historical influence remains significant.

In addition to the AT and DT, the Continuity Theory (CT) (15, 16), highlighting adaptation across the life course, argues that older adults tend to preserve long-standing patterns of behavior, preferences, and roles, and that meaning participation in later life often reflects earlier experiences and socialisation. Thus, accordingly with this theory, individuals with a history of civic or political involvement are therefore more likely to maintain such activities after retirement or in later life. Socioemotional Selectivity Theory (SST) emphasizes motivational shifts associated with perceptions of limited time. As individuals age, they prioritize emotionally meaningful relationships and activities, potentially reducing engagement in wider political or community spheres unless these activities serve emotional goals (17, 18).

The Social Capital Theory (SCT) argues that civic participation is shaped by the social capital, that, defined through bonding and bridging networks, trust, and norms of reciprocity, facilitates access to information, collective action, and civic participation (19). Thus, older adults with strong social networks and shared values may be more likely to engage in community organisations, volunteerism, and political processes.

The Life Course Perspective (LCP) adds a temporal and contextual dimension, emphasising that participation in older age is influenced by cumulative life experiences, transitions, and historical conditions (20, 21). For instance, retirement may create new opportunities for civic involvement, while cohort effects shape political orientations and participatory styles. Generational differences—for example, between pre-war cohorts and baby boomers—illustrate how sociohistorical contexts can influence engagement trajectories.

Finally, theories from political sociology, including resource mobilization and political opportunity structure, offer explanations for older adults’ high levels of political influence (22, 23). Political participation is generally interpreted as the outcome of the interaction among three main categories of factors: resources (such as economic, cultural and political capital); engagement (political interest, political knowledge, and sense of political efficacy); mobilization (social networks and interpersonal contacts that encourage participation). On the one hand, education, political skills, and income act as enabling resources that facilitate access to political participation. On the other, political interest, knowledge of the political system, and confidence in one’s own ability to influence decision-making processes are considered central to establishing a meaningful connection between the individual and the political system. In this perspective, older populations often have considerable resources—time, income stability, political experience—and are frequently supported by strong advocacy organisations such as for example trade unions retirees’ federations. These resources can enable collective action and sustained political participation (22, 23).

### 1.2 Social and political participation and the benefits for health in later life

The benefits of social participation on older adults are well documented by the literature. Older adults’ participation in social activities entails benefits for physical and mental health, and social activities that need group cohesion are even more effective, because social cohesion encourages self-improvement behaviors, indirectly promoting health (24). Participation in meaningful social roles indeed, enhances psychological wellbeing and supports a sense of purpose in later life (11). Conversely, scarce social participation is associated to a higher risk of depressive sympthoms in older adults (25). Moreover, volunteering emerges as an important health protective factor as it helps older adults counteract depression, improve self-rated health and wellbeing and increase satisfaction with life that can influence self-esteem and self-confidence (26). It can positively influence the perception of wellbeing (27), social wellbeing (i.e., quantity and quality of social relationships), improve socialisation, contrast loneliness (28) and social isolation (29).

Conversely, the effects of political participation on older adults’ health and wellbeing remains relatively limited. One of the more recent and exhaustive study is from Pavlova and Lühr (30) who conceptualise political participation as a form of agency, where older adults assert influence, express values, and maintain a role in shaping society. Political participation can benefit older adults by: maintaining a sense of purpose and agency (e.g., by remaining engaged in social issues); strengthening social connectedness (e.g., through group activities); providing cognitive and emotional stimulation (e.g., learning and discussing). The authors demonstrated that political participation can help older adults’ eudaimonic wellbeing (i.e., purpose, autonomy, meaning).

### 1.3 Policies boosting civic participation in later life: the active aging paradigm

In line with the documented benefits of social and political engagement on health and wellbeing of older adults, participation is (together with health and security) one of the three pillars of the Active Aging (AA), defined as the “process of optimizing opportunities for health, participation, and security, with the ultimate goal of improving quality of life as people grow older” (31). According with the AA framework, older adults can age healthier in presence of policy, practices, services and infrastructures that enhance physical activity, socialisation, mental health and economic and social security. AA emphasizes the importance of promoting the active involvement of older adults in work, social, economic, cultural, religious, and civic activities, to strengthen social cohesion and the sense of belonging, thereby contributing to improved mental health and quality of life among older adults.

In other words, the AA paradigm aims to put knowledge about the benefits of civic participation in later life into practice and translate it into concrete policies and measures that promote volunteering, community involvement and multi-level political participation among older adults: in neighbourhoods, cities and nations. The AA principles are implemented through the Regional Implementation Strategy (RIS) of the Madrid International Plan of Action on Aging (MIPAA) of.

the United Nations (32, 33) and they should be incorporated into national policies. The latter are operationalized with difficulties in some European Countries, due to the lack of multilevel (i.e., between European, national and regional policy) and inter-policy area dialogue, the scarce involvement of older adults in the policy processes, and the prevalence of a productivist interpretation of the AA that led Countries to concentrate on extending older adults’ working life overshadowing other AA components important for self-development, e.g., volunteering, lifelong learning, cultural activities (34).

In the following paragraph we focus on social and political participation trends in Italy and on AA policies to favour older adults’civic engagement in the Country.

### 1.4 Social and political participation in Italy

Despite social and political participation are decreasing in Italy, older adults are the age group that most contribute to the community life and interests. In 2023 (the latest data available), around 12% of people born between 1950 and 1954- that is, those in the 70–74 age group—had done so, while only 8.6% of young people aged 20–24 had engaged in at least one form of social participation in the previous year (31, 35). Generations born between 1944 and 1959 indeed, came of age in the late 1960s and 1970s, a period of major social and cultural transformation, which profoundly shaped the social activism of these cohorts even in later stages of life, right up to old age. For the same reasons, despite the voting rate in Italy passed from 86.3% in 1994 to 63.8% in 2022 in all age groups (36), the today octogenarians and septuagenarians show more interest in political issues than other age cohorts. In 2024, indeed, more than 60% of older adults aged 65–74 kept up to date with and discussed politics at least once a week, while only 48.2% of young and adult people did so (a fall of 8.9 percentage points compared with 2003) (37).

Despite the high level of social and political participation of older adults, in Italy, there is as yet no national law on AA, but a “National Plan for Active Ageing, Social Inclusion and the Prevention of Frailty in Older People” is currently being drawn up. This three-year plan forms a key pillar of Law No. 33/2023 and is to be drafted by the “Interministerial Committee for Policies in Support of the Elderly” (CIPA), established by the same law and subsequent legislative decrees (Legislative Decree 297/2024 and Legislative Decree 30/2025).

Long before the national legislative framework currently taking shape, many Italian regions had already enacted their own laws on AA, with significant disparities in the resources allocated, levels of cooperation between local authorities, associations, trade unions and citizens, and the types of measures adopted. However, beyond these differences, regional AA policies in Italy share a common denominator: the prevalence of measures designed to encourage the employment of older workers over those aimed at promoting older adults’ civic engagement (38). As highlighted by Lucantoni et al. (34), in fact, only four of Italy’s 20 regions (Emilia-Romagna, Friuli-Venezia Giulia, Umbria and Veneto), namely those with a legislative framework and implemented measures on AA, include among their objectives the strengthening of stakeholders’ participation, including older people themselves, in AA policy making.

Despite the bulk of literature on the theories of civic engagement in older age, and numerous studies on the AA legislative framework and policies, there are no studies collecting the point of view of Italian older adults’ thoughts and opinions about the benefits of social and political participation on health and wellbeing. Moreover, in aging studies, researchers often lump civic/social participation and political participation into the same positive bucket of Active Aging.

Conversely, this study wants to distinguish between the perceived effects of social vs. political participation on older adults’ health and wellbeing and provide a real-world perspective of their experience. To this purpose, the study is aimed at asnwering the following research questions: What is the experience with civic participation in a group of over 70 older adults in Italy? What is the perception of the effect of civic participation on health and wellbeing and how do social and political participation divergently impact health and wellbeing in later life?

Rather than adopting a single theoretical lens, this study utilizes these diverse perspectives as a multi-dimensional conceptual framework to analyze the varied ways Italian older adults involved in the study experience and negotiate social and political participation. In the Discussion, the empirical results are framed in the AA paradigm to reflect on how to involve older adults in existing or foreseen AA co-decisional tools.

## 2 Methods

### 2.1 Sampling, inclusion criteria and recruitment

The sample is made of 12 over 70 older adults living in a 100.000 inhabitants city in Central Italy, Ancona, characterized by one of the highest older population percentage in the Country (26.7% vs. the national average of 24.7%) (39, 35).

City’s older population is quite active. In late 2025, indeed, Ancona and its province hosted 155 voluntary and social promotion associations including also older adults, of which 14 exclusively run by older adults (eg AUSER, ANTEAS, etc.) and four cultural and recreational clubs for older people exclusively within the municipal area (Municipality of Ancona, 2026) (40). Concerning the political participation, pensioners associated to the regional branches of one of the greatest Italian trade unions, i.e., SPI CGIL, represent more than half of the total union members (41).

For these reasons, Ancona was taken as a case study, being considered representative for meaning of the Italian older adults’ experience as volunteers and activitists in the civil society at the national level.

Inclusion criteria required participants to be at least 70 years old, active in social and/or political activities in the community, and resident in the Municipality of Ancona. The first two participants contacted (IDs 1, 5) were identified from volunteers of the local branch of Caritas-Italy (i.e., the pastoral body of the Italian Episcopal Conference, established in 1971 to promote charity, social justice, and humanitarian aid across Italy and the world as part of Caritas International), while the remaining participants were recruited through non-probability snowball sampling. The fact that the first two interviewees were members of Caritas did not prevent the associative background of the rest of the sample from being differentiated; the members of this sample belonged to political parties and human rights movements and/or trade unions (IDs 2, 3, 6), Catholic Curch (IDs 4, 7) and secular/civic voluntary organisations (IDs 11, 12) (Table 1).

**Table 1:** Participants’ description.

ID	Gender	Age	Marital status	Educational level	Perceived health*	Chronic disease	Work before retirement	Type of social and/or political involvement
1	M	74	Married	Secondary school	5	No	Photographer	He always participates in every political vote; takes to the streets to demonstrate, especially on issues related to work and war; volunteers at Caritas
2	M	74	Married	Secondary school	3,5	Diabetes	White collar	He consistently votes because he believes it is important to have a political and social consciousness of the situation. He is a human rights activist especially supporting Palestine and Cuba populations
3	F	71	Married	Master degree	4	No	Practitioner	She consistently votes, viewing it as both a right and a duty. She has not changed her vote in the last two national elections (2018 and 2022). She founded an association for the rights of immigrants
4	F	86	Married	Master degree	3	Asthma	Teacher	She is not particularly interested in politics, but she always votes. Together with her husband, she mainly does voluntary work at church and in the community supporting poor and older people
5	F	76	Married	Master degree	4	No	Teacher	She has always voted, and a couple of years ago she decided to return to the streets to demonstrate against the genocide in Palestine. She started volunteering when she retired; now she teaches Italian at Caritas
6	F	72	Married	Master degree	4	No	Head teacher	She always exercises her right to vote. She is very active in social issues, is a member of both a national party and a local civic list, belongs to a trade union, is president of an association, and provides training to school staff. She is a member of the National Partisans Association
7	M	87	Married	Secondary school	4	Diabetes	White collar	He always votes and devotes most of his time to volunteering at church. He is a scout guide and also devotes his energy and commitment to transmit courting values
8	M	79	Married	Secondary school	2	No	Dock worker	He always votes, but he is discouraged by politics. In the last elections, he changed parties because he was disappointed. He does not volunteer. He would have liked to volunteer for the Yellow Cross but has always been too easily upset to do so; however, he has always been a blood donor
9	M	82	Married	Secondary school	3	Heart disease	White collar	He is very involved in both politics and social issues. He is passionate about politics, and as a result, his social activities have practical and political implications. He is part of a local civic list and has always been involved in trade union work to protect workers’ rights
10	F	83	Married	Secondary school	4	No	Housewife	She is not interested in politics and has not voted for 4–5 years, ever since her husband fell and lost much of his independence. She is involved in family support, in fact she has always been a family caregiver, first for her mother in law and now for her husband. Informal caregiving was an obstacle to social participation especially volunteering
11	F	74	Married	Secondary school	4	No	White collar	She always votes, but she expresses great disappointment with politics and politicians. Socially, she is a member of the local nematology association (AILE-Associazione Italiana Linfoistiocitosi Emofagocitica-Italian Association for Haemophagocytic Lymphohistiocytosis)
12	F	80	Widow	Secondary school	5	No	White collar	She always votes, but apart from exercising her right to vote, she shows no other interest in politics. On the social front, however, she is part of a large group of volunteers at the hospital. She founded a volunteering association

*0 = ‘very poor’ and 5 = ‘very good’.

This sampling method was adopted to gain a deeper understanding of participants’ social connections and to map the social networks fostered by their involvement in community and social activities. This approach allowed the study to access otherwise less visible relational dynamics and to expand the sample through interpersonal referrals.

One case (ID10) was intentionally retained as a negative case/contrast profile to explore the structural barriers and boundaries of non-participation (42) Including a ‘non-participating’ individual allowed us to illuminate the structural and gendered barriers, specifically, the burden of informal care in later life, that actively prevent older adult women from engaging in the public sphere. Rather than viewing participation in a vacuum, this case helped map the boundary lines between the private demands of caregiving and public civic engagement.

### 2.2 Ethics statement

Ethic Committee’s approval was not required for this study, as it asked questions on social and political participation and perceived health condition and wellbeing rather than diagnosed diseases. An informed consent was given to the individuals who accepted to be interviewed and they were asked to sign it. In the informed consent the study objectives were described and it was also specified that the participant could leave the study at any time without consequences. Data were processed in anonymized and aggregated form, in compliance with the General Data Protection Regulation (GDPR) and Italian Legislative Decree No. 101/2018. To ensure confidentiality, all collected data have been stored in a database protected by password-protected and restricted to authorized research staff only.

### 2.3 Data collection and analysis

A theoretically-driven semi-structured interview topic guide was developed specifically for this study that included questions on social participation (e.g., role and activities in volunteering organisations), political participation (e.g., electoral behavior in the two last votations, motivations to be active in politics), health self-evaluation, and the perceived effects of such a kind of civic participation on health and wellbeing. The interview ended with a question collecting suggestions for initiatives that can enhance the civic participation of older adults in order to provide evidence to policy makers for contributing to develop policy initiatives. All questions were open-ended, except for that on perceived health that asked respondents to rate their condition on a 5-point Likert scale, where 0 = ‘very poor’ and 5 = ‘very good’.

**Figure 1:**
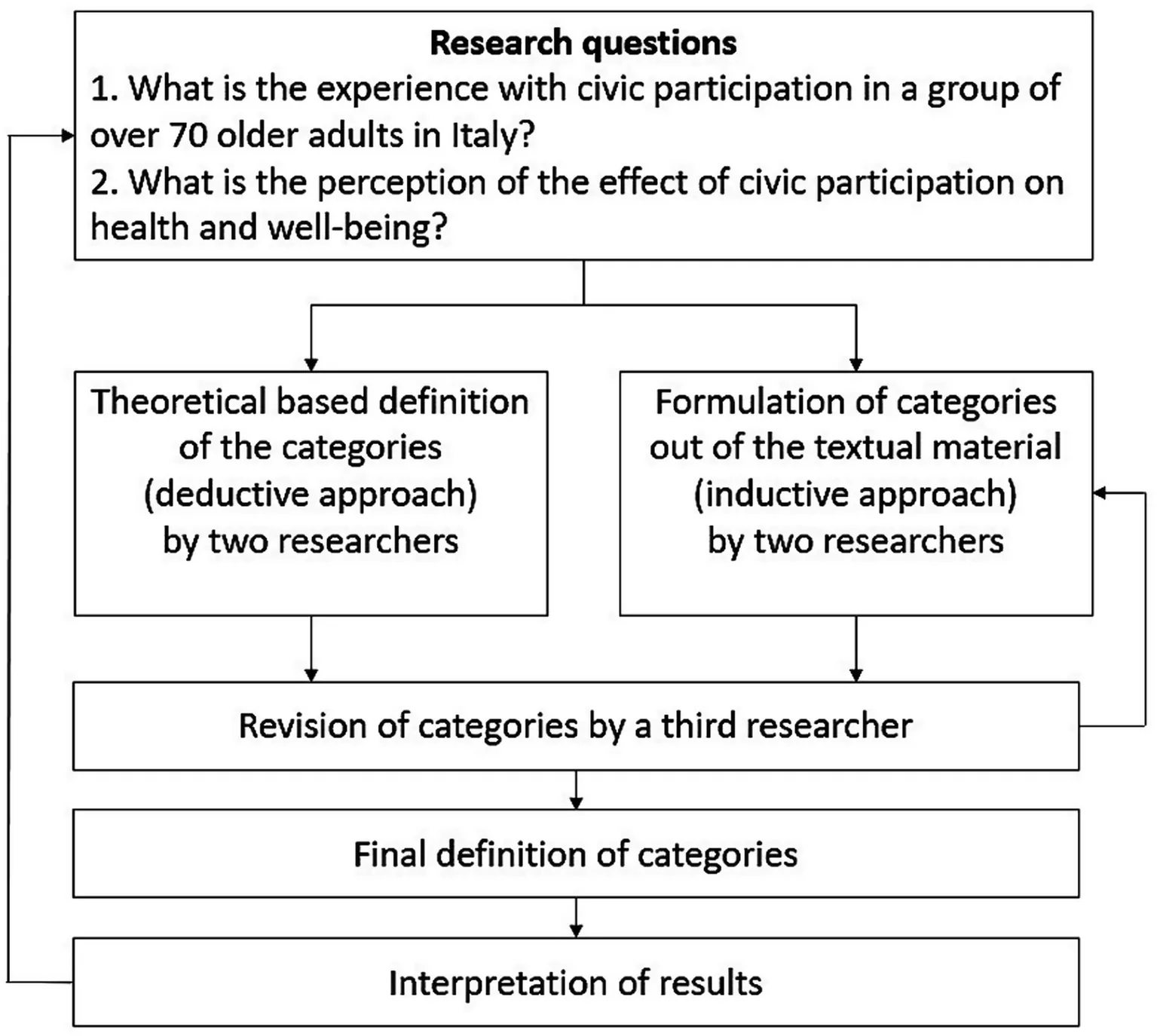
Analysis process.

Interviews were administered by a researcher with sociological background between July and October 2025. They lasted about 1 h, were audio-recorded and transcribed verbatim. Transcriptions were then analyzed by using the qualitative content analysis (43) (Figure 1), with the support of MaxQda 2024. The unit of analysis was the sentence and the textual content was divided in chunks, which were systematized in a code-tree. Then codes were associated in categories coming from the interview topics identified through the study of the theoretical frameworks on civic participation in older age (i.e., according to a deductive procedure) and categories arising directly from the interviewees’ thoughts (i.e., according with an inductive procedure). Coding was carried out by the first and the second author independently. The third author solved any disagreement in coding and finalized categories identification by helping a common interpretation of results. Because the analysis was conducted within an interpretive qualitative paradigm, the purpose of involving multiple researchers was to enhance reflexivity, credibility, and dependability through investigator triangulation and consensus rather than to achieve statistical intercoder agreement. Therefore, no intercoder agreement coefficient (e.g., Cohen’s kappa) was calculated, consistent with methodological guidance for interpretive qualitative research (42, 44–46).

## 3 Results

### 3.1 Description of the sample

The sample was made of 12 retired older adults (five men and seven women), whose mean age was 78.2 years (Table 1). Eleven participants were married and one widow. The educational level of respondents was quite high: four have a Master degree and eight a Secondary School degree. Eight respondents rated their health as good or very good and only four referred having chronic diseases. Table 1 also provides a brief description of the type of civic participation reported by the participants.

### 3.2 Categories emerged from the analysis

Five main categories raised from the deductive analysis of the textual data: 1. Thoughts on politics and society; 2. Effects of civic participation on health and wellbeing; 3. Social participation; 4. Political participation; 5. Older adults’ suggestions for improving civic participation.

Within every macro category from two to six codes were identified, raising from the words of the interviewees, i.e., according with an inductive analysis process. Table 2 reports the five macro categories, their aim and the related codes.

**Table 2:** Categories and codes.

Categories	Category aim	Codes
1. Thoughts on politics and society	To understand interviewees’ opinions on society and politics over time	Nostalgy
		Socio-economic inequalities
		Individualism
		Relationship between politics and civil society
		The predominants of economics over politics
2. Effects of civic participation (social and political) on wellbeing	To understand the effects of participation on respondents’ wellbeing	Socialisation
		Identity
		Physical health
		Mental health
3. Social participation	To understand the respondents’ social participation over time	Motivation
		Activities and role held
		Changes over time
		Specific effects of social participation
4. Political participation	To understand respondents’ political behavior and beliefs	Motivation and de-motivation of policy participation and electoral/non-electoral participation
		Political orientation over time
5. Older adults’ suggestions for improving civic participation	To collect empirical suggestions	Demonstrating
		Artistic activities for older people
		Centres and initiatives for older people to socialise
		Opinions on young people and intergenerational policies and places

In the following paragraphs we describe every category and code supported by quotations from the interviews.

#### 3.2.1 Thoughts on politics and society

The interviews reveal a complex picture of participants’ opinions on the transformations that have taken place in society and politics in recent decades. Overall, respondents express a widespread sense of nostalgy for a past that is perceived as more cohesive, participatory and less dominated by vertical power structures compared to the present.

The responses grouped under the code “Nostalgy” highlight that six participants (IDs 1, 2, 3, 5, 6, 9) think that today’s political system is characterized by an increasing hierarchisation and a growing distance between decision-makers and citizens. There is a perceived weakening of the decision-making capacity of institutions, often attributed to the “ignorance” or “incompetence” of those in positions of responsibility, and policy decisions are described as being less and less evidence-based and increasingly influenced by ideologies or partisan interests. This is seen as a clear sign of decline, observable at both the local and national levels, as clearly depicted by the following quotation:

“*Now everything is hierarchical. The obstacles are there because decisions are not made in the face of evidence, but often for ideological reasons and out of ignorance. The obstacles are there because at a certain point, when faced with the evidence, decisions are not made for ideological reasons, often due to ignorance on the part of those in charge of the system. That is the most frustrating thing. But also at the Ministry level. Especially, perhaps. No, in any case, at the local level, we have seen a worrying progressive decline”* (ID3).

Socio-economic inequalities are a further cause for concern among the respondents. For many, the growing gap between rich and poor is indicative of the distortions produced by the current model of econimic development: *This gap between the many rich and the many poor is not good* (ID11).

At the same time, some interviewees highlight a growing sense of individualism that characterises today’s society: “*today, everyone looks after their own backyard*,” says ID11 emphasising the loss of community spirit.

Concerning the “Relationship between politics and civil society,” a recurring theme concerns the transformation of the relationship between citizens, organized civil society and the political system. Several participants argue that, in the past, the presence of associations, movements and structured social groups fuelled political life, whereas today these entities have been greatly weakened. According to the interviewees, this process contributes to the “bureaucratisation” of parties and their loss of roots in society. Someone, like for example ID9, pointed out that without an adequate contribution from civil society, politics risks being reduced to mere administrative management, lacking vision to respond to social needs:*“I think civil society must bring elements into political parties.otherwise parties become bureaucratic if they are not in contact with social groups, which unfortunately have diminished compared to the past […] Ideas no longer matter, only the administrative side matters, which is bureaucratic, as if it were a permanent technical government”* (ID9).

Through quotations coded as “The dominance of economics over politics,” four interviewees (IDs 2, 3, 5, 9) denounce the growing supremacy of economics over politics, describing a “sedated” society, rendered passive by a market that seems to have assumed a central and unchallenged role. Within this context, politics is perceived as marginal and incapable of governing economic processes: “*We are in this situation of a people who are drugged because, being drugged, they do not cause problems, and everything remains in the hands of the market. The market does what it wants”* (ID2).

Some participants interpret this phenomenon as the result of the rise of neoliberalism, citing historical examples and global dynamics that, in their view, have progressively “*killed the very idea of politics*” (e.g., ID9). References emerge to the influence of economic and financial elites, technical governments and a process of progressive erosion of the decision-making role of citizens. Others, such as ID2, link the political crisis to a condition of “*no return*,” that is to say, a situation of profound decline within the political class and in political ideas themselves. In some of the interviewees (IDs 1, 2, 3), we found a general sense of outrage regarding the Palestinian issue, a topic that emerged spontaneously in response to the question about the motivations behind their social and political engagement: “Speaking of Gaza, it’s an appalling situation that I never would have expected to happen: children being killed, hospitals being targeted. Politicians these days are all hypocrites and liars; honestly, I do not know how they can stand to show their faces. I’m pinning my hopes on the streets, because the masses can shape politics. There are daily pro-Gaza demonstrations in Rome, Naples and Palermo. I hope more people will join the protests and that our leaders will decide to act against this genocide. I’ve been out on the streets myself – I’m putting my neck on the line!” (ID1).

#### 3.2.2 Effect of social participation on health and wellbeing

The analysis of the interviews highlights how the social, political and associative participation of older adults involved in the study has taken different forms throughout their lives, that were deeply significant for their overall wellbeing. The testimonies collected and reported below, show a complex picture, in which civic engagement, volunteering, membership of associations and political activities are intertwined with biographical paths, personal values, inner motivations and physical or emotional limitations.

Participation also plays an important role in identity building. Many respondents, such as ID9, describe commitment as an integral part of their being: a balance between giving and receiving to and from the community, between community usefulness and self-realisation: *“So it’s a mix between receiving, individual hope for yourself, and giving. You find yourself with other people, with companions, friends, you exchange and you are protected”* (ID9).

The most common motivation is the desire to “*do the right thing*,” to give something back, to feel useful and to cultivate values rooted in one’s own upbringing: *“You think you are making someone happy, then you realise that deep down you have done the right thing, and when you do the right thing, you feel good”* (ID4).

For IDs 4 and 12, commitment is perceived as an inner necessity, almost “*stronger than them*.” In other cases, the drive comes from political or moral awareness: ID6 talks about a civic duty, ID2 about a critical conscience that leads him not to passively accept inequalities.

*“In general, I think - and I do not think this applies to old age, but to everyone - that being committed to the common good, to a civic, political, or civic purpose, in my opinion, produces well-being. I think that unease, at least in contemporary society, is also caused by loneliness and, in some ways, selfishness”* (ID6).

There is no shortage of more personal motivations, in the perspecive of the interviews: overcoming difficult times, combating loneliness, cultivating relationships, maintaining an active routine.

Many respondents recognise that social participation also affects the body: “*Well-being is both mental and physical. The physical aspect is stupid, but it’s not bad; the weather, waking up early in the morning. I have to do it, I force myself to do it, because otherwise what’s the point? So on Thursdays and Fridays, I know that in the morning, whether it’s hot, cold, raining or not raining, I’m going down. This is physical in the sense that I wake up, then I always go an hour early, so I can go for a walk”* (ID1).

For this interviewee, it is possible to note that, for example, the simple fact of having made a commitment to the association and feeling obliged to carry out one’s duties (“*having to go anyway, whatever the weather*”) constitutes a physical discipline that stimulates an active life style.

The most significant influence identified in the narratives concerns psychological wellbeing. Most respondents describe social engagement as a source of energy, purpose, gratification and connection. For some, such as ID9, participation allows to feel alive, active, mentally stimulated and protected by networks of relationships: “*You stay alive, you always stay alive. You always have to keep up to date, just like you have to read the newspaper”* (ID9).

Daily interaction with different people, often from difficult backgrounds—such as in healthcare volunteering or reception activities—allows them to put their own concerns into perspective and build shared meanings. Many interviewees emphasise the value of relationships: feeling part of something, being recognized and recognising others, creating bonds, as reported for example by ID5: “*I have never been silent alone at home, so to speak, and so I have always tried to maintain contact with various realities, in short. However, I believe that for me it would be truly demoralising and would also diminish my balance - the goodness of my balance - not to have these contacts.”*

#### 3.2.3 Effects of political participation on health and wellbeing

In contrast to their views on social participation, where they had reported a positive perception of physical and mental health, the respondents emphasized that political participation can be a source of stress and frustration. These are said to be caused by the frequent need to manage conflict as part of political debate, but above all by the observation of a certain decline in the political class, a general lack of interest among citizens in public affairs, and a preference for individual wellbeing over the common good. This concept is clearly expressed by ID6, the same woman who declared that the “civic engagement” may produce “wellbeing”: *“Politics is also difficult; there are disagreements, arguments, misunderstandings and disappointments, so putting yourself out there inevitably leads to moments of despondency. It really gets to me, so if things do not go well, I feel terrible [.] dealing with others is sometimes difficult. It’s painful at times”* (ID6).

Similarly, ID3 told about a real feeling of “pain” for the end of the association she founded caused by the regional administration change: *“The interruption of the activities pro immigrants and the end of the association was a great source of pain for me, because, after all the work spanning almost 25 years, practically nothing remains”* (ID3).

ID2 expresses a form of “*psychological anger*” caused by the frustration coming from the prevalence of economy on politics: *“We find ourselves in a situation of no return, where there is nothing we can do, because we realise that electoral democracy is not a true democracy, simply because economic powers influence the outcome of elections”* (ID2).

What is interesting is that ID2 sayd he counters this intense anger with music: he studies and plays jazz and the blues, two musical genres that symbolise Black people’s resistance against white oppression. For him, playing music is a form of resistance.

Several barriers to volunteering and to civic participation in general also emerged: physical limitations related to age or health condition, emotional difficulties in dealing with the suffering of others, lack of time in the past, family responsibilities, and/or logistical barriers (such as lack of transport or community spaces), as underlined by IDs 12 and 10 below: “*My contribution changed in this sense, because at the beginning, if someone needed to be replaced, instead of doing volunteering one morning a week, I did three, then later with my husband, unfortunately, I couldn”t do it anymore because he needed more and more assistance”*(ID12); and *“Once I came here to the venue I am living now, I lost every social contact, with friends and associations, because this area is not connected to the city center by public transportation”* (ID10).

ID10 is a prime example of a woman who has lived for her family, who has taken on the care and support of every family member at every stage of life, as a mother, a daughter-in-law, a wife and a grandmother: “*With three children, I’ve always had plenty to do. My youngest son moved out of this house when he was 50, another when he was 40, and my 53-year-old son still lives with us. On top of that, I’ve been working. The house has always been full: now the grandchildren are here too. I looked after my mother-in-law, who had Alzheimer’s, and then my husband fell ill.”*

ID3 referred about external structural obstacles to civic participation due to the interruption of funding and support by policy makers: *“I am a doctor and, as a doctor, I founded the ‘Senza Confini’ association to promote social equality for foreign people in Italy. At the start, almost all of us were doctors and nurses, and we worked to provide healthcare for undocumented migrants and set up a clinic for them. Then there was a change of government in the regional administration; the new administration no longer supported the association and the clinic was closed down”* (ID3).

#### 3.2.4 Social participation

The analysis of the interviews reveals significant differences in the methods, motivations and impacts of social participation among the interviewees. Overall, the experiences reported show that involvement in social and voluntary activities remains a significant factor for many, albeit with changes linked to age, family circumstances and life experiences. The main motivation reported is the desire to be useful to others and to “*do the right thing*.” For several participants, e.g., ID4, the sense of having contributed to the wellbeing of others produces immediate gratification and an improvement in their own state of mind: “*Helping others and making something useful make me When I realized that I made someone happy, I know within myself that I did the right thing and this makes me at peace”* (ID4).

Most of the interviewees tell a long and continuous history of involvement in volunteering. Some, such as interviewees IDs11 and 12, describe particularly structured and long-standing experiences. ID12 has been active since 2005 and interprets volunteering as a personal need, deeply linked to her family history: “*Yes, I volunteer in nematology with AILE (Associazione Italiana Linfoistiocitosi Emofagocitica-Italian Association for Haemophagocytic Lymphohistiocytosis). It’s a wonderful association.there you see values, what values are, that is, you experience first-hand people’s suffering, and perhaps when you leave certain places you realise that everything else that is offered to us, is worthless”* and “*Giving, my need is to give, to give and to do, and volunteering addresses this need”* (ID12).

IDs1 and ID5 also volunteer regularly at Caritas, where they work two mornings a week. In this case, the experience is perceived as almost like a job, capable of strenghtening both physical and mental wellbeing, as underlined by ID1: “*When you volunteer, one thing I really want to emphasise is that it does not mean you go one day and skip the next. If you make a commitment, you have to stick to it. It’s like a job. It’s a matter of respect for the organisation and the association.”*

For other interviewees—in particular IDs 3, 6 and 9, social engagement takes on more political forms. All of them have many years of experience in activism in trade unions, associations or civic networks. ID3 has founded and led regional associations and observatories dedicated to the health and rights of immigrants. ID6 is active in political parties and anti-fascist, environmental and educational associations. ID9 participates in initiatives for the reception of asylum seeker. For these three interviewees, it is clear that volunteering is also a reason to stay constantly informed about current affairs. This entails the need and pleasure of frequently proofing social and political issues by reading articles on newspapers and specialized websites. Here below the testimony of ID3 as example:

“*Having to coordinate activities [*e.g. *for the epidemiological observatory of immigrant population founded by the interviewee and the association for the support of immigrants] and involve people [*i.e. *healthcare professionals, citizens, politicians] obviously requires you to be very well prepared and informed, because you cannot just involve people in terms of what we do, but also in terms of organisation. It gave me a lot of energy, lots of contacts, lots of ongoing relationships, even on Saturdays and Sundays”* (ID3).

Interestingly, given the advanced age of the participants, some interviewees questioned whether it would be appropriate to reduce the time devoted to activities. This decline, however, is evident only in practical terms, in relation to the frequency of protests. On the contrary, in qualitative terms of information and study, interest in social issues has not diminished: “*Not yet, it has not descreased yet, although I think that given my age, I wonder if I should continue”* (ID7).

IDs 8 and 10 stated that they were not involved in associations or volunteer activities for emotional or biographical reasons (ID8), or due to family caregiving (ID10). For example, Interviewee ID8 expressed his pleasure in volunteering with the “Yellow Cross” (“Croce Gialla”) (an ambulance service for people who are ill at home), but said he did not have the emotional resilience or energy to do it: “*Honestly, I’d like to volunteer. I would have liked to go with the “Croce Gialla,” but I’m scared. I see sick people and it hurt”* (ID8).

ID10 has never found time and energy to volunteer because she was in the front line of informal caregiving: *“No, I’ve never done any volunteer work: I’ve spent my life in the hospital because of my mother-in-law, who has Alzheimer’s [.] now I act as my husband’s ‘doctor’”* (ID10).

#### 3.2.5 Political participation

The interviewees practice political participation in two main ways: through the electoral participation (i.e., by voting to national and local elections), and non-electoral participation, i.e., through movements and/or associations built for the advocacy and defense of a particular group of people, e.g., “pro Cuba” and “pro Palestine” ones. Concerning the latter, ID2 said: *“Now there’s also the issue of Palestine, which has come to the fore in such a striking way, so I’m very sensitive to these issue”* (ID2).

Also, ID3 highlighted the importance of searching for true and reliable news in no official information channels in a time, she thinks, when information is often manipulated. In the following quotation she refers to the death of journalists in Palestine in Summer 2025: *“Today it is difficult to believe in television news. If you want reliable news you have to search fo it by surfing the Internet, but not everyone my age knows how to use the internet properly. I like to find news, read, study, deepen. For example, I am following on social media the debate on the murdered Palestinian journalists”* (ID3).

Eleven participants reported that they have always voted, while one indicated having not voted in the past four-five years (ID10), because she was too much engaged in caregiving her husband who lost most of his independence as a result of a serious accident that caused permanent damage to his back: *“I have not voted for four or five years. After he (her husband) had a fall and we were in hospital for his back operation, I lost everything, to be honest. It’s a mistake… I know… but I’m too busy… and discouraged”* (ID10).

Two motivations to vote arise from the interviews: a deep sense of duty toward the values of the democracy, and the respect of civic values introjected in the orignal family environment. In the first case, the predominant motivation to vote is a sense of responsibility towards democracy and the belief that abstention undermines active citizenship: “*Voting is a citizen’s right and duty, and as long as I have it, I exercise it. I believe it’s my duty”* (ID5).

Conversely, for ID11 and ID4, this civic obligation manifested through voting is linked to values transmitted by the family. ID11 votes not “*for a party*” but “*against a party*,” while interviewee ID8 votes “*to prevent others from deciding on their behalf*.” In general, participants noted the difficulty of finding a fully representative party; some opt for the “*least bad*” option, particularly in a context perceived as politically fragmented.

In addition to voting, several participants reported active involvement in political and social forms of participation. The interviewees IDs 1, 2, 5, 6, and 9 describe decades-long engagement in movements, associations, trade unions, and political parties.

ID6 and 9 report that their daily political activity includes organizing events, attending assemblies, and supporting international causes. ID1 expresses political participation also through public demonstrations, while ID5 links her activism to trade union traditions and the belief that protest is an essential element of democracy: “*Political life as active participation is voting, but another form of active participation is demonstrations. I believe that peaceful demonstrations are a sign of democracy, a way of expressing dissent towards something that can be improved. This has been my approach since I joined a trade union as a recent graduate and pursued social and economic demands, among others, that I could assert in my work”* (ID5).

IDs1 and 3 emphasise that taking to the streets is also a way to build “critical mass”: *“It is important to take to the streets because a large crowd can influence the government’s decisions. The larger our numbers, the more change we can bring about”* (ID1); and *“I understood that we need to build a critical mass”* (ID3).

All interviewees did not change the structural orientation of their vote during their participation process. IDs1 and 7 maintain that during an election they changed parties, but always within the same area, as depicted by the following quotation: *“Yes, there was a change. I changed, obviously to the center-left. I consider it closer to me, even if it’s not very powerful, but it’s in the coalition”* (ID1).

#### 3.2.6 Suggestions for improving older adults’ civic participation

Participants highlighted the perception of a growing need to propose concrete recommendations and suggestions to improve the civic participation of older people. In particular, from the point of view of the interviewees, it is essential to reach a critical mass of people involved, as individual interventions are not considered sufficient. The value of collective action is considered central to generating meaningful change: “*I realized that we need to reach a critical mass. Critical mass, yes, that is, mass above all else, because here it seems to me that we are so much more than individuals”* (ID3).

One respondent also expressed interest in artistic activities designed for older people, perceived as useful tools for promoting wellbeing, personal expression, socialisation and participation in older age: “*With theatre, for example, imagine how many older people could perform theatre and do it very well”* (ID5).

The narratives clearly highlight the importance of centres and initiatives dedicated to bringing older people together. Participants emphasized the lack of informal spaces where they can meet and contrast loneliness—such as dance halls, places to play cards or recreational areas—and consider this absence to be an obstacle to social life: “*I feel that many people around me are lonely. Perhaps we should dedicate ourselves to helping these people”* (ID4).

Many interviewees (e.g., IDs 1, 3, 5, 6) expressed a positive view of young people and underlined the need of educating them about political participation, as proved by the following quotations: *“I see hope above all in young people like you, because there are young people who are dedicating themselves to education, university and the environment. It is right that we older people involve young people in volunteering and politics. Young people do not vote and cannot get involved because the right conditions aren’t created, such as meeting places”* (ID3), and *“We should teach young people the importance of voting, because the tick on the ballot paper is our only weapon”* (ID1).

The interviewees also showed a significant interest in projects and places that encourage intergenerational encounters and bi-directional learning, as underlined by IDs 5 and 6: *“We need places where young and older people can meet and discuss politics”* (ID5); and *“My suggestion is for young and older people to do an activity together, and then perhaps it will become clear that older people do not always know more than young people. We older people are always a bit inclined to tell young people what to do, but it can also be the other way round. Debating with my daughters often puts me to the test, because they actually know so much more than I do. I realized that people younger than me, partly through the media, manage to form an opinion on current affairs much more quickly than I do”* (ID6).

While showing a certain detachment towards activities reserved exclusively to older people, participants appreciated initiatives that promote the sharing of experiences and common activities (e.g., courses, workshops). In addition, they expressed the desire that these spaces should not be limited to recreational activities, but also include opportunities for discussion and debate on policy, social, and current issues. According to respondents, the lack of such opportunities contributes to maintaining mutual stereotypes between young and old and creating “closed systems” devoid of intergenerational dialogue: “*Being among older people does not appeal to me at all. I appreciate the activities promoted by older people’s volunteer organisations, that try to offer something that brings older people together to do and share things together (*e.g.*, handcraft, cook, sewn). But I do not like them. I would like places where people meet, perhaps to take a cooking class or an English course, etc., to also have spaces for discussing and debating the problems of society and the world. Otherwise, what comes out of it? Among older people, they say, “Ah, these young people.” and so there is no. it’s all a closed system” (ID6).*

## 4 Discussion

The study aimed to contribute to the scientific debate on the experience and meanings attributed by older adults to social and political participation, by focusing on the results coming from interviews with 12 over 70 older adults living in a medium-sized city in Central Italy. The first novelty of the study lies in the age of the target, focused on individuals aged 70 and over, considering that previous studies focused generically on over 50 individuals, as highlighted by Akhter-Khan et al. (47). The second novelty is the fact that the study has been carried out in a time when it looks like the political awareness, understood as striving towards the “res publica,” i.e., the common good, seems to have been reawakened by the “pro-Palestine” protests and demonstrations that swept across Italy between June and November 2025. All generations were united for a common issue, for the first time following many years of political apathy among young people, disinterest among adults and disillusionment among older adults, mirrored by the decreasing voting rate (36). Finally, the study helps designing an empirical view of the civic participation in later life by distinguishing the divergent effects of social vs. political participation on older adults’ perceived health and wellbeing.

In the following sub-paragraphs, we interpret the results in light of the previous ad available scientific literature by recalling the two study research questions.

### 4.1 What is the experience of respondents with civic participation in a group of over 70 older adults in Italy?

Participants’ social and/or political participation most mirrors the same patterns they had when they were younger, in accordance with the Continuity Theory (15, 16). Nevertheless, the study enriches the current knowledge by showing that although the civic activity level of some “older old” respondents decreased over time—i.e. in terms of the frequency with which they attend meetings and/or take to the streets to demonstrate, due to physical limitations related to advancing age -their contribution in terms of political thought remains alive and rich in civic, moral and ethical values. This wealth of experience should be valued and exploited through intergenerational learning programmes, for the benefit of both younger generations, who are increasingly less inclined to participate in political and social life and interact with older adults, and of older ones who might see recognized their life-long participation effort.

On the one hand, framed in the Socioemotional Selectivity Theory (17, 18), the interviewees engaged in politics in youth remained active even in older age and did not prefer voluntary, despite disappointment with today’s Italian politics. This finding leads us to believe that older adults may continue to engage in community organisations and politics despite the fact that this can sometimes be a source of frustration and “pain,” because they derive pleasure and satisfaction from the human connections and relationships forged during their social and political activism, rather than from the ideology that has shaped the action itself. In other words, these relationships may fulfil their need for emotional fulfilment. Alternatively, remaining politically active, despite disappointments and frustrations, may depend on the tendency of some older adults to recall and process positive events more easily than negative ones, as observed by Carstensen et al. (48).

### 4.2 What is the respondents’ perception of the effect of civic participation on health and wellbeing? How do social and political pariticipation divergently impact health and wellbeing in later life?

A core contribution of this study lies in highlighting the stark asymmetry between social and political participation in later life. While literature often clusters these under the umbrella of civic engagement, our findings reveal divergent health pathways. Volunteering operates as a powerful mechanism for Active Aging (31): it fosters self-efficacy, self-esteem, and social connectedness (11, 25–29). Crucially, participants emphasized that formal volunteer commitments create a strong sense of accountability and purpose. This “duty of care” translates directly into physical activation, motivating older adults to structure their days and leave the house even during low-motivation states.

Conversely, political participation presents a far more ambivalent, and at times detrimental, health outcome. Based on the interviewees’ testimonies, political participation may be a source of frustration and anger for someone who no longer identify with the current way of doing politics in Italy and complain about the absolute influence of economics and finance on political choices that ideally should be oriented towards the common good. In line with Pavlova and Lühr (30), political participation can be a form of agency with positive outcomes on the cognitive functions because the debate may be a helpful stimulus for the brain, in terms of logic and language. Neverthelss, this study highlights that political participation may also be detrimental for the emotional and psychological health of some older adults. This is in line with recent literature (49, 50), asserting that political participation may not be as beneficial for the individuals involved as non-political participation, e.g., volunteering, because attempts to exert political influence lead to intergroup conflict and unmet goals, and are sometimes viewed as morally reprehensible and self-serving. Thus, the benefit of political participation may have been undermined by the current political climate in Italy.

The study also shows the extreme vitality of the older people interviewed and their desire to improve the communities in which they live thanks to their past experience and their current involvement in voluntary associations, parishes, political movements and neighbourhood committees.

It is important to emphasise that both volunteering and political engagement are meaningful for older adults and that this meaning gives purpose to the lives of mature individuals. In other words, as Erikson states (51), civic engagement (social and/or political) allows older adults to express their generativity, defined as a concern for establishing, nurturing, and guiding the next generation expressed through activities contributing productively to society. Successful resolution of this stage leads to a sense of purpose and care, whereas failure results in stagnation and self-absorption (30, 52).

In line with the above, it is worth mentioning that respondents expressed the need for more frequent interaction with young people through physical spaces for sharing. Exclusive interaction with peers appears sterile and stagnant, and does not serve to change the status quo or foster new ideas that could improve today’s society, in which the older respondents no longer seem to recognise themselves: they would line a society that should be less individualistic, more oriented towards the common good, and more supportive.

The inclusion of ID10, a woman who has never being engaged in social nor in political activities, in the analysis allowed to reflect on gender inequalities in civic participation in all life ages. ID10, in fact, first took care of three children who stay very long time at home with parents (one was till living with parents in his fifties), then she helped her mother in-law living with Alzheimer’s Disease, and finally she was caring for her husband with long-term care needs. Moreover, the fact that ID10 has not voted for at least 4 years, confirms the predominance of the private sphere (that of the home, intimacy and family ties) over the public sphere (characterized by civic engagement, the desire to learn about and understand the events of the historical era in which one lives, and the desire to influence its course) for this woman.

This case can be framed in the literature that highlights that opportunities for AA are deeply constrained by structural gender inequalities in Italy, first and foremost, the extensive involvement of women in providing ongoing care for the most vulnerable members of their families, e.g., children and older adults with disabilities (53). This divide is rooted in cultural norms, specifically a strongly fragmented familistic welfare regime, and structural market disadvantages reflecting in pension gender gap (54).

Other barriers to civic participation are living in peripheral areas with limited transports (e.g., ID10), health issues (e.g., ID12), and the lack of physical places to meet not so much with older peers as with young people (ID6), who are considered by respondents to be a source of inspiration and essential interlocutors for the personal growth of mature people.

The interviews in fact, reveal a very positive view of young people and trust them, who are considered not only the future but, beyond all rhetoric, mainly the present of the Country. At the same time, older interviewees express extreme distrust in this and previous Governments for the lack of vision and policies to support young people and increase intergenerational understanding and solidarity.

### 4.3 Study limitations, research suggestions and policy recommendations

This is a qualitative study based on an Italian purposive small sample. It therefore does not claim to be exhaustive in terms of the meanings attributed by Italian senior citizens to social and political engagement nor of effects of participation on mental and physical health of older adults. The small sample did not allow to interpret data in a gender perspective. Nevertheless, the results may be useful for better understanding how over 70 older adults may represent a lever for change, if they are engaged in dialogue with the younger generations. This result can add a piece to the available literature on the civic participation of older adults and pave the way for further studies aimed at testing how intergenerational dialogue can change older and younger individuals’ perspective on social and political engagement and verifying whether young people are equally interested in such exchange and in physical, rather than virtual, spaces where this can take place.

Further studies focusing on the effects of civic participation on different health realms are welcome to understand if it may have adverse effects on older individuals’ health, when it is source of frustration and anger. Future studies with larger samples should also verify gender differences in the level of participation, health outcomes and meanings attributed to social and political participation, what has been not possible to do by this study, due to the very small number of interviewees. Furthermore, more studies are needed for investigating the possible correlation between informal caregiving and political disengagement in a gender perspective, the motivations to remain engaged in politics in later life despite frustration, verifying the effects of different types of social and political engagement on older adults’ physical activity and understanding how much such activities can contrast sedentary and help the prevention of cariovascular diseases, as intuited by Wu et al. (55).

Concerning policy recommendations, local administrations are called to favouring intergenerational political dialogue and social cooperation by providing places easily accessible for older as well as young people and guaranteeing public transports and connections. Digital literacy educational programmes may multiply the chances for meeting and discussing issues that are of common interest for all the generations.

At the European level, policy frameworks aimed at promoting Active Aging must avoid a one-size-fits-all approach to civic engagement. Promoting voluntary social participation directly yields health and mobility benefits through routine and social utility. However, expecting older adults to find wellbeing through formal political channels without addressing systemic alienation may inadvertently exacerbate emotional distress and frustration.

In Italy, according to the clustering of Italian Regions with similar AA policy and initiatives, development made by Lucantoni et al. (34), the frustration and emotional stress or older adults engaged in politics, could be addressed by strengthening the presence of older and younger people in the regions where AA co-decisional tools already exist (e.g., in Emilia-Romagna and Friuli-Venezia-Giulia); create co-government tools where they do not yet exist (e.g., Puglia, Basilicata, Lazio), and make a list of stakeholders prioritizing older adults and young people in regions with weak AA policies and interventions (e.g., Lombardia and Sicilia). Intergenerational stakeholder consultation forums can, in fact, serve as spaces where young and older people can gain a better understanding of their respective rights and responsibilities, and where better AA interventions can be designed.

Furthermore, the narrative of ID10 suggests that to build truly effective and fair AA policies in Italy, future strategies must tackle gender-based divides as fundamental determinants of health and wellbeing.

## 5 Conclusion

The present study aimed to explore the experience of social and political participation among individuals aged 70 and over and to understand how they perceived the effects of civic engagement on their health and wellbeing.

Through the testimonies collected from 12 older adults living in Central Italy, the study sought to address two main research questions: how civic participation is experienced in later life and what perceived health outcomes are associated with such participation.

The findings show that civic participation represents, for many respondents, a stable and meaningful component of their personal and social identity. Most participants maintained forms of engagement that had already been present in their youth, confirming the importance of biographical continuity in social and political involvement. In some cases, retirement also created new opportunities for participation, allowing older adults to devote more time to volunteering, community associations, and civic activism. Social and political participation therefore emerged not as a marginal or residual activity of old age, but as a central dimension through which older adults continue to exercise agency, civic responsibility, and a sense of community belonging.

The interviews also highlight how civic participation is generally perceived as beneficial for health and wellbeing. In particular, volunteering and social engagement appear to promote the maintenance of an active lifestyle, support mental health, counteract loneliness, and strengthen feelings of social usefulness and self-esteem. Respondents described associative involvement as a source of daily motivation that encourages them to leave the house, maintain meaningful relationships, and feel like an integral part of the community. Social participation therefore appears to be closely linked to psychological, relational, and even physical wellbeing. In contrast, political participation showed more ambivalent effects. On the one hand, it helps older adults maintain critical, cognitive, and reflective capacities and provides a sense of continuity with the values developed throughout their lives. On the other hand, it may generate frustration, anger, and feelings of powerlessness in the face of a political context perceived as distant, bureaucratized, and dominated by economic interests. Indeed, respondents expressed deep disappointment with contemporary politics, while still considering voting and public participation to be fundamental elements of democratic citizenship. This finding suggests that the effects of political participation on wellbeing may also depend on the political climate and the level of trust in institutions and democratic processes.

One of the most relevant aspects emerging from the study concerns the value attributed to intergenerational dialogue. Respondents did not wish to be confined to spaces exclusively dedicated to older people; rather, they called for places and opportunities for interaction with younger generations, based on mutual exchange, shared learning, and joint participation in social and political life. Participants perceived younger generations not merely as recipients of civic values passed down by older adults, but also as active interlocutors capable of contributing fresh perspectives, new skills, and meaningful cultural insights. In this sense, civic engagement can be interpreted as a form of generativity, through which older adults continue to contribute to society and transmit experiences, values, and collective memory.

The findings highlight how older adults can represent a fundamental resource for democratic and community life, not only through volunteering and social activism, but also through the transmission of experiences and civic values to younger generations.

Promoting civic participation among older adults therefore means not only supporting their individual wellbeing, but also strengthening social cohesion, intergenerational solidarity, and the quality of democracy. In this regard, intergenerational programmes, spaces for public discussion, shared cultural initiatives, and social inclusion policies may represent strategic tools for enhancing the contribution of older adults to contemporary society and for counteracting both social isolation and the growing civic disengagement observed in aging Western societies.

## Data Availability

The raw data supporting the conclusions of this article will be made available by the authors, without undue reservation.
